# An Overprotective Nose? Implicit Bias Is Positively Related to Individual Differences in Body Odor Disgust Sensitivity

**DOI:** 10.3389/fpsyg.2020.00301

**Published:** 2020-02-28

**Authors:** Marta Zuzanna Zakrzewska, Marco Tullio Liuzza, Torun Lindholm, Anna Blomkvist, Maria Larsson, Jonas K. Olofsson

**Affiliations:** ^1^Gösta Ekmans Laboratory, Department of Psychology, Stockholm University, Stockholm, Sweden; ^2^Department of Surgical and Medical Sciences, “Magna Graecia” University of Catanzaro, Catanzaro, Italy; ^3^Department of Psychology, Stockholm University, Stockholm, Sweden

**Keywords:** olfaction, disgust, implicit bias, behavioral immune system, authoritarianism, body odor disgust sensitivity

## Abstract

Body odors are universal elicitors of disgust, a core emotion that plays a key role in the behavioral immune system (BIS) – a set of psychological functions working to avoid disease. Recent studies showed that body odor disgust sensitivity (BODS) is associated with explicit xenophobia and authoritarianism. In the current experimental pre-registered study (https://osf.io/6jkp2/), we investigated the association between olfactory pathogen cues, BODS and implicit bias toward an outgroup (tested by an implicit association test). Results show that BODS is positively related to implicit bias toward an outgroup, suggesting that social attitudes may be linked to basic chemosensory processes. These attitudes were not influenced by background odors. Additionally, BODS was related to social, but not economic conservatism. This study extends the BIS framework to an experimental context by focusing on the role of disgust and body odors in shaping implicit bias.

## Introduction

### Disgust and Prejudice

Is there a link among odor disgust, disease-avoidance mechanisms, and prejudice? Disgust is a basic emotion and a key aspect of disease avoidance. It is crucial for the behavioral immune system framework (BIS, Schaller, 2006) that comprises a set of behaviors whose purpose is to avoid encountering pathogens. Because fighting a disease is costly for the body, false-negatives (neglecting an infected object) can be more dangerous than false-positives (avoiding a harmless object). Therefore, BIS can be activated by both illness-related cues and those false-alarms that only resemble (superficially) the actual symptoms of pathogen infection (Schaller and Park, 2011). Like a fire alarm setting off when we cook, disease-avoidance responses can be triggered by non-contagious cues, such as physical, moral or sexual deviations. For example, BIS and feelings of disgust have been associated with stigmatization of ethnic (Navarrete and Fessler, 2006) and sexual minorities (Inbar et al., 2012), xenophobia (e.g. Faulkner et al., 2004), opposition to immigrants (e.g. Aarøe et al., 2017), and other forms of prejudice (see Terrizzi et al., 2013 for meta-analysis). Moreover, disgust sensitivity has been linked to political attitudes such that higher levels of disgust sensitivity are related to political conservatism (Inbar and Pizarro, 2016).

### The Role of Olfaction and Body Odors in Avoiding Disease

Olfaction allows us to detect invisible – but smelly – pathogen cues (Stevenson, 2009). Body odors are particularly salient cues and universal triggers of disgust (Curtis and Biran, 2001). Humans can use these body odor cues to detect signs of disease and regulate social behaviors accordingly (Olsson et al., 2014). Despite evidence suggesting that human olfaction, and body odors in particular, might play an important role in navigating the social world (e.g. Sorokowska et al., 2011; de Groot et al., 2017) human olfactory disgust has received relatively little attention from researchers. For example, olfactory disgust has played a minor role in previous assessments of disgust sensitivity, such as the Disgust Scale-Revised (DS-R, Olatunji et al., 2007) and the Three Domains of Disgust (TDDS, Tybur et al., 2009), with the number of olfactory-related items ranging between 5 and 16%. To fill this gap, we recently developed and validated the Body Odors Disgust Scale (BODS, Liuzza et al., 2017), a 12-item scale that describes scenarios involving six different body odors (sweat, breath, feet, gas, urine, feces) coming from internal and external sources. As compared to those other assessments, BODS is more strongly correlated with perceived vulnerability to disease (PVD, Duncan et al., 2009), a result that supports the notion that body odor perception might be more relevant in detecting pathogen cues and activating the appropriate behavioral response (Liuzza et al., 2017). Higher disgust sensitivity to body odors has been related to higher explicit bias toward a fictive refugee group (Zakrzewska et al., 2019) showing that individual differences in BODS levels are interesting in the context of prejudice.

### The World as a Dangerous and Pathogen Rich Place

Differences in levels of disgust sensitivity are not the only individual differences in personality traits that have been linked to both ideology and prejudice. The dual process framework (Duckitt, 2001) proposes two distinct processes underlying individual differences in prejudice. The first process involves perceiving the social world as a dangerous place, which prompts authoritarian ideology; the other involves viewing the social world as a “competitive jungle,” which is associated with social dominance. These processes may fuel negative attitudes toward immigrants, because authoritarianism may be viewed as a form of avoidance (preserving existing cultural norms, traditions and ‘old-fashioned values,’ e.g. Hainmueller and Hiscox, 2010) and social dominance is linked to aggressive competition (a response to increased economic and job competition, e.g. Sniderman et al., 2004). Both authoritarianism and social dominance are consistently and positively correlated with prejudice (Sibley and Duckitt, 2008). They also correspond to distinct personality traits; while authoritarianism is linked to low openness to experience, social dominance is linked to low agreeableness (e.g. Sibley and Duckitt, 2010; Cohrs et al., 2012). A previous study (Liuzza et al., 2018), showed that BODS relates more to authoritarianism than to social dominance or endorsement of social inequality. Authoritarianism might be considered more closely linked to avoidance and, similarly to BODS, may be conceptualized as a defense against possible pathogen threats (Oaten et al., 2011; Osmundsen and Petersen, 2016). Incorporating the dual process framework into BIS research can help understand the relationships between different factors that contribute to prejudice, such as odor disgust sensitivity and authoritarian views.

### Quantifying Prejudice

Today, in many Western societies, overt racism is disapproved of, and people are often unwilling to report negative attitudes toward ethnic or racial minorities. To counter a possible response bias induced by such impression-management in explicit self-report measures, an increasing number of prejudice researchers use implicit measures of social cognition (Fazio and Olson, 2003).

The implicit association test (IAT; Greenwald et al., 1998) is one of the most widely used implicit measures of stereotypes and prejudice (Greenwald et al., 2009). The IAT is a categorization task in which reaction-time differences between prejudice-congruent and prejudice-incongruent trials (divided by participants’ variability) provide a standardized measure of bias (D scores). The IAT appears to be a fairly reliable measure of implicit bias toward social outgroups, whether based on race, sexual orientation, gender, or political preference (Nosek et al., 2007; but see Bar-Anan and Nosek, 2014 for a recent evaluation). The IAT could be used to address the hypothesis that implicit associations might underlie xenophobic and authoritarian attitudes previously shown in disgust-sensitive persons.

### Body Odor Disgust, Disease, and Implicit Prejudice – The Present Study

In the present study, we wanted to extend the evidence for the role of body odor disgust in BIS by investigating implicit bias toward a stigmatized minority out-group in Sweden, namely the Romani people, hypothesizing that such bias would be positively associated with BODS scores. Moreover, we tested whether the presence of an unpleasant body-like odor would activate disease-avoidance concerns and thus boost the implicit bias toward the outgroup. Of particular interest was to investigate if people with higher BODS levels display a steeper increase in their implicit bias when exposed to an unpleasant body-like odor as compared to a neutral or pleasant odor. As we assume that prejudice can be partially explained by disease-avoidance concerns, we predicted that our hypothesized effects would be stronger when using an alternative version of the IAT with health/illness related words (see Bulsing et al., 2009). Additionally, we hypothesized that authoritarianism mediates the relationship between the BODS and implicit bias, thus repeating the pattern of relationship from Liuzza et al. (2018). We pre-registered detailed hypotheses on OSF.

## Materials and Methods

### Preregistration

Materials, procedure, hypotheses and planned analyses were preregistered on the Open Science Framework Repository (OSF^1^). To test the preregistered hypotheses more directly, we performed additional model comparisons, which were not stated in the preregistration.

### Participants

We recruited participants according to Sequential Bayes Factor design (Schönbrodt and Wagenmakers, 2017). We defined a minimal sample size of 20 and planned to keep recruiting participants until we found enough support either for the null (BF_01_^2^ = 3) or for the alternative hypothesis (BF_10_ = 6). A BF of 6 for the alternative hypothesis was chosen because (a) there is a known asymmetry in the sample size necessary to gain enough evidence for the null vs. the alternative (Schönbrodt and Wagenmakers, 2017) (b) the choice of the default prior effect size distribution parameters in the *BayesFactor* package can change the BF by a factor of 2 (Schönbrodt et al., 2017). Thus, a BF of 6 would ensure that, even in the worst scenario, evidence in favor of the alternative hypothesis would be at least moderate. As we had time and economic constraints in collecting data, we planned to stop sampling whenever we reach a sample size of 70. This maximum sample size was determined through a Null Hypothesis Significance Testing *a priori* power analysis (power = 80%) for a medium effect size (*r* = 0.3) and a one-tailed hypothesis. We used a one-tailed hypothesis because we predicted a positive relationship between BODS levels and implicit bias, and a negative relationship would go against our hypothesis. We ended data collection with a final sample size of 35 participants.

The sample consisted of Swedish-speaking, healthy (no self-reported nasal, neurological or psychiatric disorder) participants who took part in the experiment in exchange for course credit or gift vouchers. The sample included mostly females (*n* = 25) and had a mean age of 28.5 years (*SD* = 6.29). Participants were informed about possible unpleasantness of certain smells in the study.

The study was carried out in accordance with the recommendations of and approved by the Swedish Ethical Review Authority. All subjects gave written informed consent in accordance with the Declaration of Helsinki.

### Measures

Using the web-based software Qualtrics, participants filled out a self-paced survey containing several scales and questions about age, education, and gender. The set of scales provided an assessment of explicit prejudice. Questions about hygiene concerns and olfactory abilities were collected for exploratory purposes and are not reported in this paper.

#### Right-Wing Authoritarianism (RWA)

We used a validated version of the RWA with 15 items that did not refer to specific minority populations and hence avoided conflating authoritarianism with specific prejudice (Zakrisson, 2005). Participants reported their level of agreement with each statement (e.g. “The ‘old-fashioned ways’ and ‘old-fashioned values’ still show the best way to live.”) on a seven-point scale ranging from 1 (*totally disagree)* to 7 (*totally agree*). The scale showed a high internal consistency (Cronbach’s α = 0.8). Individual RWA scores were computed by averaging the answers for all items, after reversing the score of the inverted items.

#### Social Dominance Orientation (SDO)

We used a well-established 16-item version of the SDO, which measures the tolerance for inequality among social groups (Pratto et al., 1994). Participants were asked to indicate how they felt about each of the 16 statements (e.g. “Some groups of people are simply inferior to other groups.”) on a 1 (*very negative*) to 7 (*very positive*) Likert-type scale. The scale showed a very high internal consistency (Cronbach’s α = 0.93). Individual SDO score was computed as the mean of all answers, after reversing the score of the inverted items.

#### Social Distance Scale (SDS)

We used a modified version (Müller et al., 2014) of the original SDS (Emory, 1933). Participants were given four scenarios (‘Please imagine that this person lives in your neighborhood’) followed by a description of a person who is a high school student and engages in a religious community in the free time. This person could be either male or female and either Swedish or Romani. Participants had to rate how they would feel if such person (a) was their closest neighbor, (b) took care of their parents, (c) married into their family. Ratings were provided on a four-point scale, from *very positive* (1) to *very negative* (4). The scale showed an excellent internal consistency (Cronbach’s α = 0.95). Individual SDS scores were computed as a mean of answers for all items. Detailed information about responses on the SDS questionnaire is available in Supplementary Table S2, including percentages of participants who replied positively or negatively to each scenario.

#### Body Odor Disgust Sensitivity Scale (BODS)

The 12-item scale measures disgust sensitivity to six types of body odors (upper body sweat, feet, feces, urine, breath, and gas; Liuzza et al., 2017), each of which appears in two different contexts: internal (e.g. “You are alone at home and notice that your breath smells strongly”) and external (e.g. “You are sitting next to a stranger and notice that their breath smells strongly”). In each of the 12 smell contexts, participants rated to what extent the scenario elicits disgust on a Likert type of scale ranging from 1 (*not disgusting at all*) to 5 (*extremely disgusting*). The scale showed a very high internal consistency (Cronbach’s α = 0.93). Individual BODS score was computed as the mean of all answers.

#### Implicit Association Test (IAT)

**FIGURE 1:**
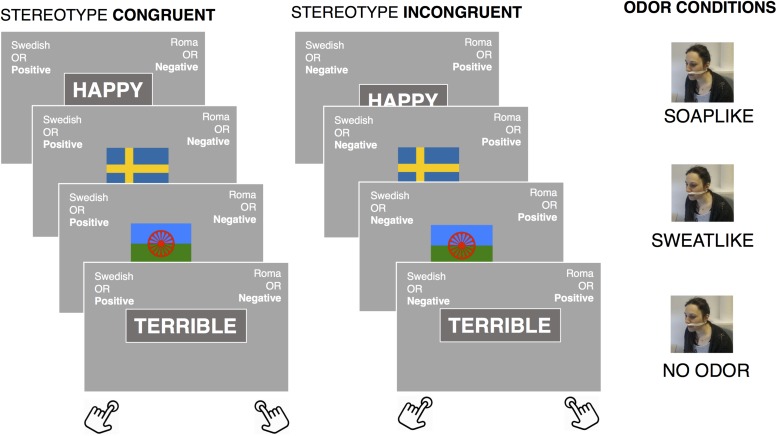
Experimental procedure. This figure illustrates an example of trials in the normal IAT, in both congruent and incongruent blocks. The BIS IAT was identical, with the exception that we used health and sickness related words. Participants were instructed to classify the words and pictures as quickly as possible. IATs were presented in pairs: each pair consisted of both IATs (BIS and normal) and was repeated three times: with a soap-like odor, a sweat-like odor, and a neural odorless cotton pad. The order of odor conditions was randomized between participants.

Later, participants performed the remaining parts of the study including the IAT (Greenwald et al., 1998). The IAT involves categorization of words and pictures, with eight stimuli per category. The words were categorized as *pleasant* or *unpleasant* and the pictures were categorized as representing (the in-group) *Swedish* or (the out-group) *Romani* culture. The pictures were of neutral valence and covered eight areas: man, woman, children, family, housing, newspaper, flag, and folklore dress. Two different versions of the IAT were administered in a counterbalanced order: (1) a standard set of the IAT valence-words, (2) a BIS-related version, where we used words associated with disease, e.g. “disease,” “health,” “rotten,” “fresh,” etc. Participants were requested to respond (categorize) as quickly and accurately as possible to the stimuli that appeared on the monitor. The entire procedure consisted of five blocks: 1 (bad vs. good/illness vs. health) and 2 (Swedish vs. Romani) were single categorization blocks that helped participants to familiarize with the task and learn which stimuli belong to which category. Blocks 3–5 were combined categorization tasks in which participants had to press the same keys to categorize Romani-related pictures or negative/illness words vs. Swedish-related or good/health in the (congruent block), or Swedish or negative/illness words vs. Romani or positive/health words (incongruent block). Block 4 served the purpose of changing from incongruent to congruent (or vice versa). Each block consisted of 32 trials. The stimuli were presented in random order within each block. Figure 1 illustrates the experimental procedure with examples of congruent and incongruent trials.

The association strength was determined by computing the standardized difference between the response times in the incongruent vs. congruent block. Data from these blocks was used to compute a difference score, following Greenwald et al. (2003) with an error penalty method.

### Odor-Conditions

Both versions of the IAT were administered under three different odor-conditions: unpleasant and sweat-like odor (valeric acid), pleasant and soap-like odor (lilac), and a no odor condition (clean cotton pad). In previous research, valeric acid has been used as human feet sweat-like odor (Anderson et al., 2003; Syrjänen et al., 2017). Both odors were diluted with an odorless mineral oil solution (Propylene glycol, 1.2-propanediol 99%, Sigma-Aldrich) in order to reach what equals a moderate intensity. Based on our previous psychophysical investigations, a 30% concentration corresponds to a moderate intensity for both odors (Syrjänen et al., 2017). Participants were exposed to odors via cotton pads placed in tube-shaped cotton bands, tied under their noses – a method that has proved efficient in previous studies (Syrjänen et al., 2017, 2019). All three odor-conditions were manipulated within participants. As a control measure, participants had to rate intensity and hedonic valence of the odors (see Supplementary Figure S1). The rating was made on a seven step, Likert-type scale, ranging from not detectable to extremely intense (intensity), and from extremely unpleasant to extremely pleasant (hedonics).

Participants were first instructed verbally on how to use the IAT-test, and then they read instructions at the beginning of each IAT condition. In each odor condition, participants performed the two versions of the IAT, in order to (a) reduce the time required to change the cotton pads (b) minimize the possible after-effects of the exposure in one odor condition to the subsequent one. The order of odors and IAT versions was randomized between participants, yet the order of the two IAT versions was the same across odor conditions. After completing the IAT sessions, participants were thanked, debriefed, and received their compensation.

### Missing Data

Due to technical issues, four participants underwent two of the same versions of IATs and four of the other (instead of three of each), nevertheless we kept their data. One participant did not answer to one SDS question; we used the remaining answers to calculate the SDS score.

### Data Analysis

We conducted a linear multilevel modeling (LMM) analysis (Pinheiro and Bates, 2000) in order to consider individual level (ID) sources of random variability in the intercepts. Unlike traditional statistical methods, LMM is suitable for (a) accounting for the non-independence of observations with correlated errors; (b) separating the effects caused by the experimental manipulation (fixed effects) and other effects (random effects).

We tested our hypotheses within a Bayesian hypothesis-testing framework using Bayes Factors (BFs) and the *BayesFactor* package (Morey and Rouder, 2015). Specifically, we tested the main effect of odor exposure (unpleasant vs. pleasant vs. neutral), of IAT version (standard vs. BIS-related) and the moderating roles of the BODS. We added the order of blocks within each IAT (Congruent-incongruent vs. incongruent-congruent) to the model to control for block order effect on the D scores. We used measures of social and economic conservatism to test their possibly mediating role. We interpreted and labeled the sizes of BFs according to the recommendations of Raftery (1995) as referred to by Jarosz and Wiley (2014).

In the preregistration we stated that we would select the best model using the *generalTestBF* function from the *BayesFactor* package. This function allows to compare a set of defined models and pick the best one. As it does not allow to test each of the pre-registered hypotheses directly against a corresponding null hypothesis, we decided to additionally model our hypotheses using the *lmBF* (linear model Bayes Factor) function from the same package, using nested models, in order to compare the evidence for each preregistered hypothesis against a corresponding null. For all models, we used the default prior (Cauchy distribution left at its default value: *r* = √2/2). Following the current standards, we use subscripts on BFs to refer to the models begin compared. Accordingly, the BF for the alternative relative to the null is denoted BF_10_, while the BF for the null relative to the alternative is denoted BF_01_. We used the *BayesMed* R package (Nuijten et al., 2015) for the mediation analysis (*Hypothesis 1c* below) – we did not specify this package in the preregistration. A detailed description of the models used to test each hypothesis can be found in Supplementary Material.

#### Hypotheses

The hypotheses were pre-registered on OSF^3^ before data collection:

– The IAT will show that respondents (Swedish adults) will display a negative implicit association bias toward Roma. (*Hypothesis 1a*)
– Respondents that score higher on BODS will show a more negative bias toward Roma people than respondents scoring low. (*Hypothesis 1b*)
– We expect hypothesis 1b to be mediated by measures of social conservatism (RWA and SDS) but not economic (SDO) conservatism. (*Hypothesis 1c*)
– Exposure to the unpleasant “body odor” will strengthen the negative implicit associations toward Roma people, compared to the control conditions (no odor, and pleasant lilac odor). (*Hypothesis 2a*)
– Respondents scoring higher on BODS will show a more marked increase in negative implicit associations in the “body odor” condition than people scoring lower. (*Hypothesis 2b*)
– We expect that the results anticipated in hypotheses 2a and 2b will be stronger in the BIS IAT as compared to the standard IAT. (*Hypothesis 2c*)

#### Corresponding Models

We tested our hypotheses in two ways: first, we used *generalTestBF* (pre-registered analysis), which formulates models including all combinations of specified effects, compares these models to a model with intercept only and orders them according to the relative likelihood of the data conditional on these models vs. the intercept-only model. Thus, we specified all effects relevant for our hypotheses: BODS, OrderInIAT, IATtype, Odor, and a three-way interaction between Odor, IATtype and BODS. Additionally, we specified ID as a random effect which should always be kept in the model.

Second, we tested our hypotheses in a more direct way: by building models which represent each of the hypotheses and comparing these models against corresponding null models (which did not include the predicted effects). Table 1 includes model formulation of each hypothesis and the corresponding null model. This direct model comparison has not been preregistered, however the reader should note that the models included the same exact effects as those specified in the preregistration.

**TABLE 1:** Hypotheses and corresponding models.

Hypothesis	Model		Model notation
Hypothesis 1a		Model 1a:	Dscore ~ 1 + OrderInIAT + ID
Hypothesis 1b		Model 1b:	Dscore ~ BODS + OrderInIAT + ID
		*Null model:*	Dscore ~ 1 + OrderInIAT + ID
Hypothesis 1c	Mediation path	RWA	RWA ~ 1 + BODS
		SDO	SDO ~ 1 + BODS
		SDS	SDS ~ 1 + BODS
Hypothesis 2a		Model 2a:	Dscore ~ OrderInIAT + Odor + ID
		*Null model:*	Dscore ~ 1 + OrderInIAT + ID
Hypothesis 2b		Model 2b:	Dscore ~ BODS + OrderInIAT + Odor + BODS:Odor + ID
		*Null model:*	Dscore ~ BODS + OrderInIAT + Odor + ID
Hypothesis 2c		Model 2c:	Dscore ~ BODS + OrderInIAT + IATtype + Odor + BODS:IATtype + IATtype:Odor + BODS:Odor + BODS:IATtype:Odor + ID
		*Null model:*	Dscore ~ BODS + OrderInIAT + IATtype + Odor + BODS:IATtype + IATtype:Odor + BODS:Odor + ID

*Dscore – IAT difference score, OrderInIAT – the order of the IAT combined tasks (Congruent-incongruent vs. incongruent-congruent), Odor – effect of odor exposure (disgusting vs. pleasant vs. neutral), IATtype – version of the IAT (standard vs. BIS related), BODS – body odor disgust sensitivity score, RWA – right wing authoritarianism score, SDO – social dominance orientation score, SDS – social distance score. Additionally, all models included a random effect of participant (ID). Note that model 1a was used as the null model for hypotheses 1b and 2a. All models are formulated in Wilkinson notation.*

We started by testing Hypothesis 1a (Model 1a). We followed the model up by sampling from the posterior distribution of the intercept with 10000 iterations in order to assess if zero was included in the 95% credible intervals, which would speak against bias present in our sample. Next, we compared model 1b (Hypothesis 1b) with a corresponding null model (not including BODS). We planned to test the hypothesis of mediating effect of RWA and SDO on the relationship between BODS and D score, provided that there is evidence for the correlation between the variables and mediators. We checked these requirements by comparing the mediation path models against a model with intercept only (no BODS; Hypothesis 1c). To test if implicit bias was affected by odors (Hypothesis 2a), we compared model 2a with a corresponding null model (not including Odor, Table 1). In order to see if there is an interaction between the effect of Odor and BODS (Hypothesis 2b) we tested model including all main effects and an interaction effect (model 2b) against a model without the interaction (Table 1). Lastly, to test if this interaction effect is different in the BIS and normal IAT, we compared a model including the main effect as well as all two-way interactions (between IAT version and BODS, IAT version and Odor, BODS and Odor) with the model including also the three-way interaction between BODS, IAT version and Odor (Hypothesis 2c).

For all the model comparisons, we first assessed if the model representing a given hypothesis is better than the null model. If yes, then to interpret the strength and the directions of the observed effects we sampled from the posterior distributions of each model with 10000 iterations.

### Additional (Not Preregistered) Analysis

#### Authoritarianism, Social Dominance, and BODS

We decided to integrate data obtained in this study with data from another study investigating the BODS – RWA and BODS – SDO relationships (Liuzza et al., 2018). Specifically, we wanted to update the results from Liuzza et al. (2018) with data collected in the current study, to get a meta-analytical estimation of the effect (details in Supplementary Material). This analysis was not preregistered as the other dataset was not available at the time of preregistration. Such integration of results enabled us to focus on the estimation of the effect rather than the BF and see if outcomes from this experimental study were consistent with those of the previous, and much larger, survey.

## Results

Descriptive statistics for BODS, RWA, SDO, and the IAT scores are available in Table 2.

**TABLE 2:** Descriptive statistics for scores on all four scales and the IAT.

		Mean estimate
BODS		3.21 (0.82)
RWA		3.14 (0.77)
SDO		1.81 (0.79)
SDS		2.05 (0.62)
D score	IAT normal	0.37 (0.3)
	IAT BIS	0.43 (0.32)

*The numbers in brackets represent standard deviations.*

### Preregistered Analysis

#### BODS Is Related to Implicit Bias

The model including BODS and the order of blocks within the IAT emerged as the best model. BODS was included in the best six models (out of the total of 17 compared models, nine contained BODS). When comparing the first model excluding BODS with the winning model, we found very strong evidence in favor of the winning model (BF_10_ = 315). Table 3 shows all models and the BF corresponding to comparison of each model vs. an intercept only model (a model assuming that all effects are 0). Residuals (observed – predicted values) in the winning model were normally distributed and homoscedastic (see Supplementary Figure S2).

**TABLE 3:** Best model selection.

Model		BF
Dscore ∼	BODS + OrderInIAT + ID	–
	BODS + OrderInIAT + IATtype + ID	2.72
	BODS + OrderInIAT + Odor + ID	13.58
	BODS + OrderInIAT + IATtype + Odor + ID	38.67
	BODS + ID	87.34
	BODS + IATtype + ID	248.01
	
	OrderInIAT + ID	314.90
	BODS + Odor + ID	622.07
	BODS + OrderInIAT + IATtype + Odor	713.93
	+ BODS:IATtype:Odor + ID	
	OrderInIAT + IATtype + ID	880.25
	ID	973.53
	IATtype + ID	2560.43
	BODS + IATtype + Odor + ID	3175.64
	OrderInIAT + Odor + ID	3864.55
	OrderInIAT + IATtype + Odor + ID	11347.10
	Odor + ID	12281.22
	IATtype + Odor + ID	32681.35

*All models (in Wilkinson notation) used for selecting the best model, and the BF corresponding to a comparison of each model with the winning one (displayed in the top row of the table). The dashed line marks the first model without BODS.*

### Direct Model Comparisons

#### BODS Is Related to Implicit Bias

**FIGURE 2:**
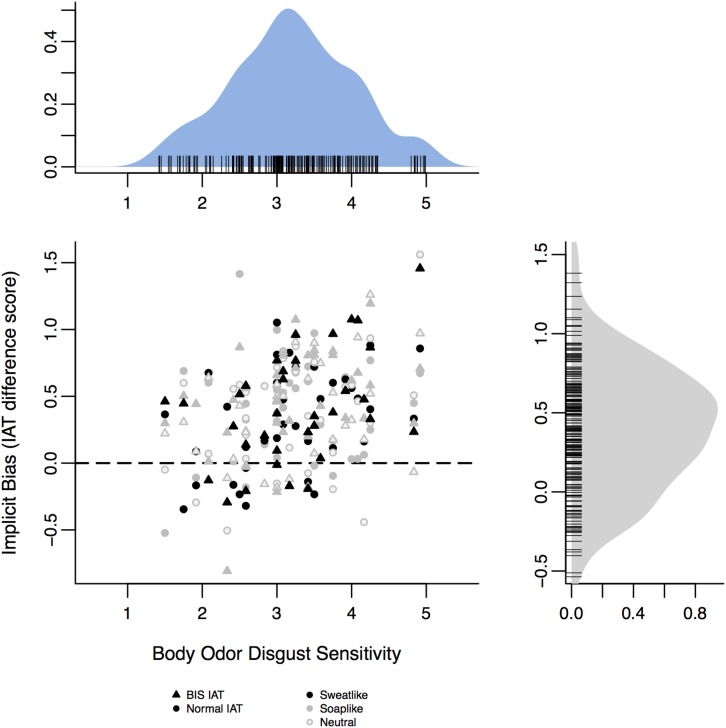
Body odor disgust sensitivity is positively related to implicit bias. Positive IAT difference scores indicate bias against the outgroup, while negative values indicate bias against the ingroup. No bias (0) is marked by the dashed line. The upper and right panels depict the density distribution of BODS and IAT D score respectively.

Through the additional direct hypothesis testing, we found a small-to-medium size negative bias toward the outgroup in our sample (mean D score = 0.40, 95% posterior credible intervals (PCI) = [0.30, 0.49]). Furthermore, we found very strong evidence in favor of a positive relationship (mean posterior estimate = 0.17, 95% PCI = [0.09–0.28]), between BODS and implicit bias against Roma people (Hypothesis 1b, BF_10_ = 315.72, Figure 2).

#### Odor Exposure and IAT Version Are Unrelated to Implicit Bias

We found evidence in favor of no relationship between odor exposure and implicit bias (Hypothesis 2a, BF_01_ = 15.31). Similarly, there was no interaction between odor exposure and BODS (Hypothesis 2b, BF_01_ = 105.59) and the evidence spoke against any role of IAT version (Hypothesis 2c, BF_01_ = 21.5, BF_01_ = 4.76 without Odor). Mean estimates for the effects of odor and IAT version were close to 0 (ranging from −0.03 to 0.03, all PCIs including 0) and can be found in the (Supplementary Table S4).

### BODS Is Related to Measures of Social Distance, But Not Economic Conservatism

**FIGURE 3:**
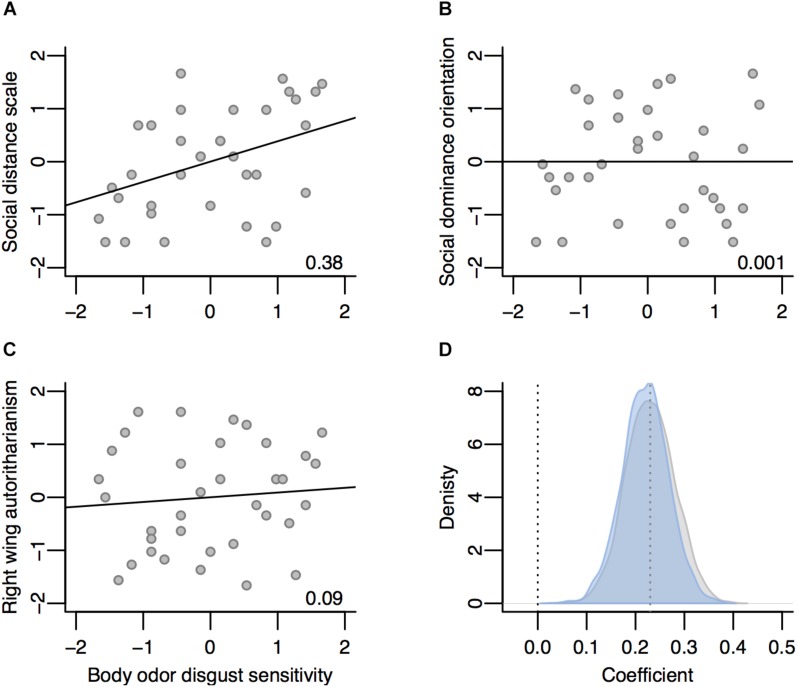
The relationship between BODS and SDS **(A)**, BODS and SDO **(B)**, and BODS and RWA **(C)**. All variables were ranked and standardized. Numbers in the lower right corner of each plot are the regression coefficients. Blue distribution on **(D)** is the posterior distribution of the estimate for BODS – RWA coefficient and the gray one is the prior – based on Liuzza et al. (2018). Hence, although the correlation is not supported by the BF in our sample, the estimates from combined evidence from both studies remained relatively stable. Dotted lines represent 0 (no relationship) and 0.23 – the lowest coefficient from Liuzza et al. (2018) (more details about this analysis are available in Supplementary Material).

BODS was positively associated with SDS (mean posterior estimate = 0.38, 95% PCI = [0.07–0.71], BF_10_ = 6.9, Figure 3A) but SDS did not mediate the relationship between BODS and IAT score (mean posterior estimate = 0.08, 95% PCI = [−0.05 to 0.26], BF_01_ = 3.37). There was no relationship between BODS and SDO (mean posterior estimate = 0.005, 95% PCI = [−0.31 to 0.31], BF_01_ = 3.1, Figure 3B). There was no notable relationship between BODS and RWA, however the evidence was inconclusive (mean posterior estimate = 0.09, 95% PCI = [−0.21 to 0.4], BF_01_ = 2.58, Figure 3C). Therefore, we discarded the two corresponding mediation analyses.

### Secondary Analyses

#### BODS Is Related to RWA but Not SDO

After updating the BODS – RWA relationship coefficient from previous study (Liuzza et al., 2018) with the current data, the new 95% PCI [0.13, 0.32] still did not include 0 and there was evidence against the null relationship (BF_10_ > 1000). This outcome favors the notion of an existing, positive relationship between the two constructs (Figure 3D). In contrast, after updating the BODS – SDO relationship, the new 95% PCI [−0.11 0.07] included 0 with a BF_10_ < 1, pointing to no relationship between BODS and SDO.

## Discussion

In the current preregistered study, we found that disgust sensitivity to body odors (BODS) is related to implicit bias toward an outgroup. This result corroborates and extends previous findings about the relationship between BODS, a BIS-related measure (Liuzza et al., 2017), and attitudes toward outgroups (Zakrzewska et al., 2019). These findings strengthen the view that some individuals may have a more sensitive BIS which makes them prefer behaviors and attitudes that limit contact with out-groups.

Contrary to our hypothesis, implicit bias was not influenced by a background body-like unpleasant odor. We need to point out two possible drawbacks of the paradigm used in our study. First, the odors were presented constantly over the duration of an experimental block. This might have resulted in habituation (Smeets and Dijksterhuis, 2014) to the smell, and suppression of its effect, although similar studies in our lab has shown no evidence of habituation (Syrjänen et al., 2017, 2019). To confirm the null effect of odors (or to find evidence for its existence), further research is needed where odor cues are brief and paired with each target stimulus (as they are in a priming task) rather than serving as background odors. Research shows that congruency effects can be observed on a single trial level through priming, also in the olfactory modality (e.g. Olofsson et al., 2014; Kastner et al., 2016). Second, we did not use actual body odors but odors that resemble them. However, valeric acid is present in body odor, especially in disease (Pandey and Kim, 2011; Shirasu and Touhara, 2011), and the substance has been widely used as sweat-like odor in other studies (e.g. Anderson et al., 2003; Jacob et al., 2003). What may be of more importance is that the smell was not presented in a way suggesting that it came from another person. Again, we believe that a cue – target priming design could help reveal the influence of odor on group biases. In hindsight, although we did not find any odor effects, we think that similar studies would benefit from having not only a body-odor related unpleasant odor, but also another, non-bodily yet unpleasant odor, thus allowing to talk more directly about body odor disgust.

Even though the theoretical framework emphasizes the role body odors and body-odor related disgust we would like to acknowledge a potential limitation of our study in drawing conclusions about the effect of body odors. Namely, BODS is the only measure of disgust we used. While comparing BODS with other disgust measure (such as DS-R or TDDS) we could have potentially shown an olfaction-specific link between disgust sensitivity and implicit bias. However, previous study (Liuzza et al., 2017) showed that BODS is correlated with both DS-R and TDDS (0.37 > = *r* < = 0.65) while at the same time being more strongly related to PVD than to the other measures, pointing to the relevance of body odor disgust sensitivity (BODS) for pathogen avoidance. Thus, we believe that although including another measure of disgust could have strengthened our claims, we would not have gained much more valuable information.

Faulkner et al. (2004) showed elevated BIS activation and IAT bias for a task evoking danger (rather than general unpleasantness), yet we did not find this effect for our disease-related IAT. In fact, the two versions of the IAT were highly correlated in the current sample. Since health/illness-related words are also strongly valenced, the lack of task version effect might be explained by a difficulty to differentiate between the strength of the target group to a health/illness concept, vs. the target group to a positive/negative concept associations, especially given the relatively small sample.

Lastly, our results should be viewed in the light of the dual process framework. We could not replicate our previous findings on US samples (Liuzza et al., 2018), where we found a stable, small-to-medium relationship between RWA and BODS. It is possible that our sample, including Swedish college students, is not representative of all ranges of authoritarian attitudes (only two observations fell above the theoretical midpoint of the RWA scale). However, it should be noted that, when comparing nationally representative samples and convenience samples, Aarøe et al. (2017) found almost identical correlation coefficients for the association between individual differences in disgust sensitivity and social attitudes. By including these new data to update previous observations from Liuzza et al. (2018), we found that the previous positive relationship between BODS and RWA is still credible. Additionally, current results show a relationship between BODS and another measure of social conservatism, namely the SDS. These relationships can be explained in the light that RWA, SDS and BODS can be related to avoidance of possible pathogen threats, in the world perceived as a dangerous place. Similarly to Liuzza et al. (2018) we found no relationship between BODS and a measure of social dominance (SDO), which refers more to the perception of the world as a competitive jungle and is thus connected to other sources of prejudice, not related to disease avoidance.

Taken together, this study extends current knowledge about odor disgust and BIS with evidence suggesting that social attitudes may be linked to basic chemosensory processes.

## Data Availability Statement

The datasets generated for this study can be found in the Open Science Framework repository (https://osf.io/6jkp2/).

## Ethics Statement

The studies involving human participants were reviewed and approved by Swedish Ethical Review Authority. The patients/participants provided their written informed consent to participate in this study. Written informed consent was obtained from the individuals in the figures for the publication of any potentially identifiable images or data included in this article.

## Author Contributions

MTL, TL, and JO developed the study concept and design. MZ contributed to data collection. MZ and MTL performed the data analysis and drafted the manuscript, with the help of AB, TL, ML, JO who provided critical revisions. All authors approved of the final version of the manuscript for submission.

## Conflict of Interest

The authors declare that the research was conducted in the absence of any commercial or financial relationships that could be construed as a potential conflict of interest.

## Footnotes

1 https://osf.io/nbwe5/

2 BF_01_ represents support for the null hypothesis (over the alternative) while BF_10_ shows support for the alternative hypothesis over the null hypothesis.

3 https://osf.io/6jkp2/
