# Supplementary material for: An Overprotective Nose? Implicit Bias Is Positively Related to Individual Differences in Body Odor Disgust Sensitivity
Source: Front Psychol. 2020 Feb 28;11:301. doi: 10.3389/fpsyg.2020.00301 (PMC7059856; doi:10.3389/fpsyg.2020.00301)
Supplement: Supplementary file 1 [file Data_Sheet_1.pdf]

Supplementary Material

1. Methods

1.1 Additional (pre-registered) analyses

1.1.1 Sensitivity analysis for the effect of BODS on implicit bias

In the pre-registration, we state that we will use the default prior (Cauchy distribution left at its default value: $r = \sqrt{2}/2$) but that we will test the consistency of results by using other ($r = 1/2$ and $r = \sqrt{2}/2$) parameters. Thus, we run the winning model (including the effects of BODS, order within the IAT, and a random effect of ID), and null model (including only the effect of order within the IAT and a random effect of ID) with these other priors.

1.2 Additional (not pre-registered) analyses

1.2.1 Right-wing Authoritarianism, Social dominance orientation and BODS

We decided to integrate data obtained in this study with data from another study investigating the BODS – RWA and BODS – SDO relationships (Liuzza et al., 2018). Specifically, we wanted to update the results from Liuzza et al. (2018) by data collected in Study 3, in order to get a more meta-analytical overview of the effects. We chose Study 3 as it contained all the three variables. Unfortunately, previous studies did not have data on SDS, thus we were unable to perform similar analysis for the BODS – SDS relationship.

The following analysis has not been preregistered due to the fact that we did not have (or know if we will have) the data at the time of preregistration. We find this analysis necessary, especially considering some inconsistencies of results from the current and the previous study.

In order to do parameter estimation, we first ranked the BODS, SDO and RWA scores in order to have a robust statistic of the relationship between these measures and the BODS. Next, we used the *brms* package (Bürkner, 2016) to do a linear regression with custom priors based on Study 3 from Liuzza et al. (2018. For the RWA analysis we specified the prior on the estimate as a normal distribution with a mean of .23 (the value of the smallest relationship between RWA and BODS in Liuzza et al., 2018) and a standard deviation of 0.05, which corresponds to the difference between: the median, and the 2.5% quantile divided by 2 (data available at OSF: https://osf.io/c8xjy/). Analogously, for the SDO analysis we specified the prior on the estimate as a normal distribution with a mean of −0.02 and a standard deviation of 0.05. Lastly, we looked at the 95% posterior credible interval to see if it includes 0.

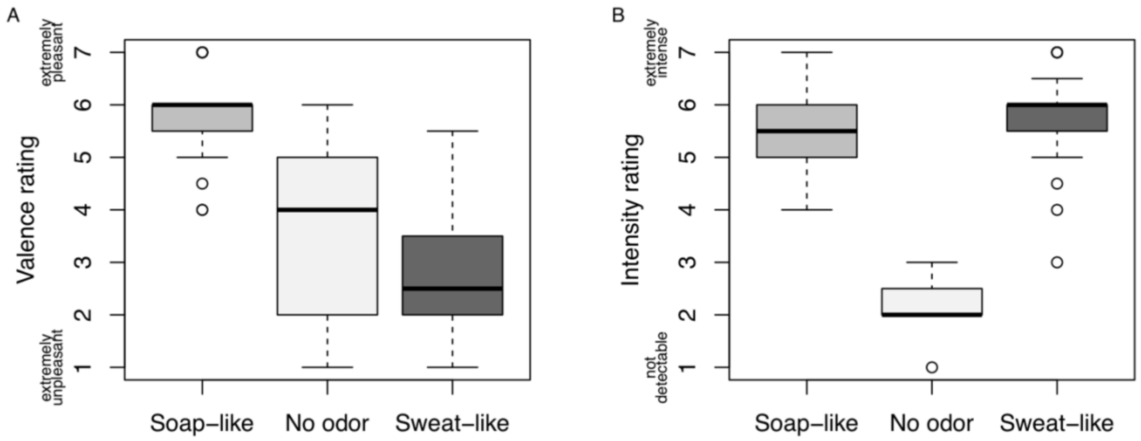

Fig S1. Odor ratings. Boxplots of mean valence (A) and intensity (B) ratings for odors used in the study.

1.2.2 Valence ratings

When designing the study, we assumed a priori that valeric acid will be perceived as unpleasant and lilac as pleasant. Although the odors seem to be rated according to our assumptions (Figure S1), to control for the possibility that the odor manipulation did not work due to differences in odor valence perception, we performed an exploratory analysis using valence ratings made by participants. We did this by replacing the odor category (`Odor`) in models corresponding to Hypothesis 2a and 2b with valance ratings for each smell:

Hypothesis 2a_2:

Perception of valeric acid odor as unpleasant will strengthen the negative implicit associations towards Roma people, compared to the control conditions (no odor, and pleasant lilac odor).

We compared model 2a_2 with a corresponding null model (not including the odor valence rating – `OdorValenceRating`, see Table S1).

Hypothesis 2b_2:

Respondents scoring higher on BODS will show greater change in negative implicit associations for condition with odors perceived as unpleasant, than people scoring lower on BODS.

In order to see if there is an interaction between the effect of odor valence rating (`OdorValenceRating`) and `BODS` we tested model including all main effects and an interaction effect against a model without the interaction (see Table S1).

Hypothesis	Model	Model notation
Hypothesis 2a_2	Model 2a_2:	`Dscore ~ OdorValenceRating + OrderInIAT`
	null model:	`Dscore ~ 1 + OrderInIAT`
Hypothesis 2b_2	Model 2b_2:	`Dscore ~ OdorValenceRating:BODS + OdorValenceRating + BODS + OrderInIAT`
	null model:	`Dscore ~ OdorValenceRating + BODS + OrderInIAT`

Table S1. Hypotheses and corresponding models. Abbreviations used in the models: `Dscore` – IAT difference score, `OrderInIAT` - the order of the IAT combined tasks (Congruent-incongruent vs incongruent-congruent), `OdorValenceRating` – effect of the perceived valence of the odors, `BODS` – body odor disgust sensitivity score. All models included a random effect of participant (`ID`).

1.2.3 Social distance scale

Table S2 shows details about attitudes towards the different scenarios in the SDS questionnaire.

	…be your neighbor		…care for your parent		…marry family member		Total	
	Swedish	Roma	Swedish	Roma	Swedish	Roma	Swedish	Roma
Very positive	28.9	25.0	35.5	29.3	17.1	19.7	27.2	24.7
Rather positive	56.6	53.9	50.0	44.0	44.7	35.5	50.4	44.5
Rather negative	13.2	21.1	11.8	20.0	34.2	34.2	19.7	25.1
Very negative	1.3	0.0	2.6	6.7	3.9	10.5	2.6	5.7
positive	85.5	78.9	85.5	73.3	61.8	55.2	77.6	69.2
negative	14.5	21.1	14.4	26.7	38.1	44.7	22.3	30.8

Table S2. Detailed results from the SDS scale. Numbers represent percentage of participants who chose each of the four options for the scenarios in the SDS questionnaire. 'Positive' and 'negative' rows include aggregated scores for the two positive, and two negative options respectively.

2. Results

2.1 Sensitivity analysis for the effect of BODS on implicit bias

The estimates were almost identical no matter the prior used in the analysis. The BFs for the comparison of the BODS model with the null (no BODS) model, were also very similar (all BF_{10} > 100, Table S2).

		mu			BODS estimate		
**prior**	**BF_{10}**	**Mean**	**95% PCI**	**SD**	**Mean**	**95% PCI**	**SD**
medium	106.397	0.403	[0.325, 0.479]	0.039	0.183	[0.081, 0.286]	0.052
wide	109.247	0.404	[0.327, 0.481]	0.039	0.182	[0.08, 0.282]	0.051
ultrawide	109.433	0.404	[0.327, 0.479]	0.039	0.182	[0.077, 0.281]	0.052

Table S3. Sensitivity analysis with different priors. Mean estimates for the intercept (**mu**) and BODS effect (**BODS estimate**) and their corresponding 95% PCI intervals and standard deviations (**SD**). We provide also the bayes factors in favor if the model with BODS vs. the null model (no BODS) (**BF_{10}**). Medium prior was a Cauchy distributuion with $r = \sqrt{2}/2$, wide prior was a Cauchy distributuion with $r = 1$, and the ultrawide prior was Cauchy distributuion with $r = \sqrt{2}$.

2.2 Odor exposure, as well as IAT type, is unrelated to implicit bias

Table S3 includes estimates for the effects of odor and IAT type, tested in Hypothesis 2a and 2c. Note that all estimates were close to 0 (ranging from -0.03 to 0.03, all PCI including 0).

		Mean estimate [95% PCI]
**Odor**	pleasant body-like odor	0.02 [-0.03 0.08]
	unpleasant sweat-like odor	-0.02 [-0.08 0.03]
	neutral (no odor)	0 [-0.06 0.05]
**IAT**	normal	-0.03 [-0.07 0.1]
	BIS	0.03 [-0.01 0.07]

Table S4. Estimates for odor and IAT type effects. Estimates for effect of odor (Hypothesis 2a) and IAT type (Hypothesis 2c). The estimates reflect deviations from average. Note that all PCI's include 0.

2.3 Valence of odors are not related to implicit bias

We found evidence in favor of no relationship between odor valence and implicit bias (Hypothesis 2a_2, mean posterior estimate = 0.01, 95% posterior credible intervals (PCI) = [-0.02, 0.03], BF_{01} = 4.32). There was no interaction between odor valence and BODS (Hypothesis 2b_2, BF_{01} = 4).

2.4 Model diagnostics for the winning model

Below we visualize the distributions of the residuals (observed – predicted values) in the winning model, as well as chain diagnostics for the predictors included in the model.

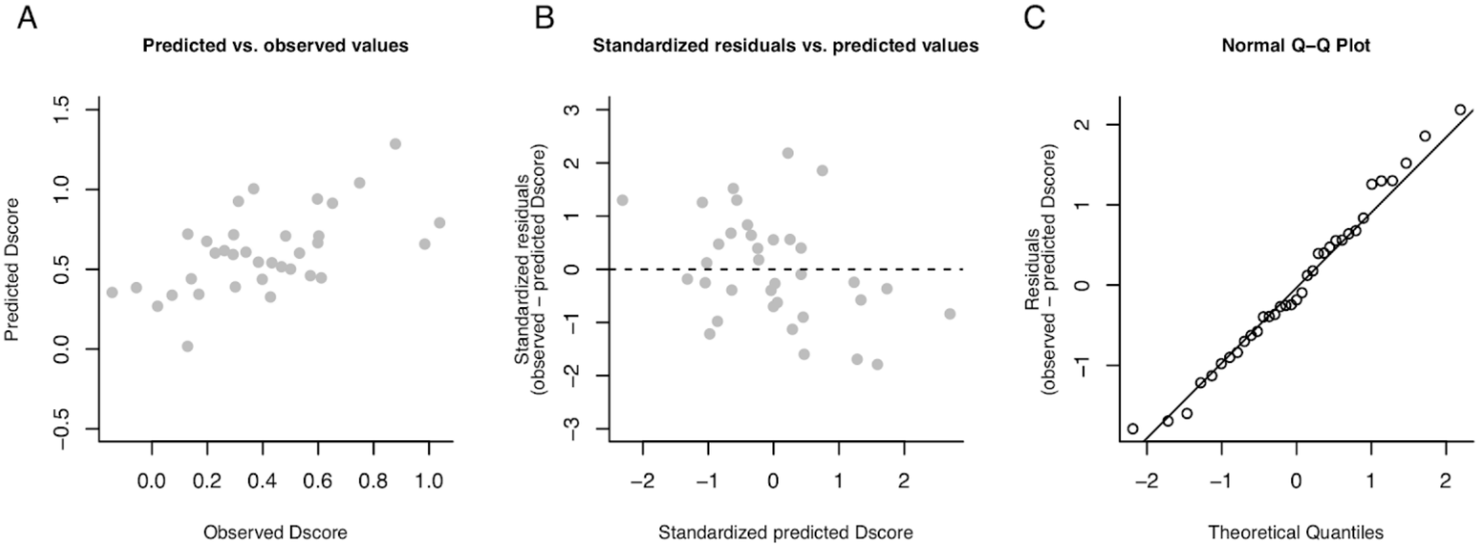

Fig S2. Residual plots for the winning model. Panel A shows predicted D score for each observed D score. Based on plots B and C we concluded that the assumptions of homoscedasticity of residuals (B) and their normal distribution (C) were met.

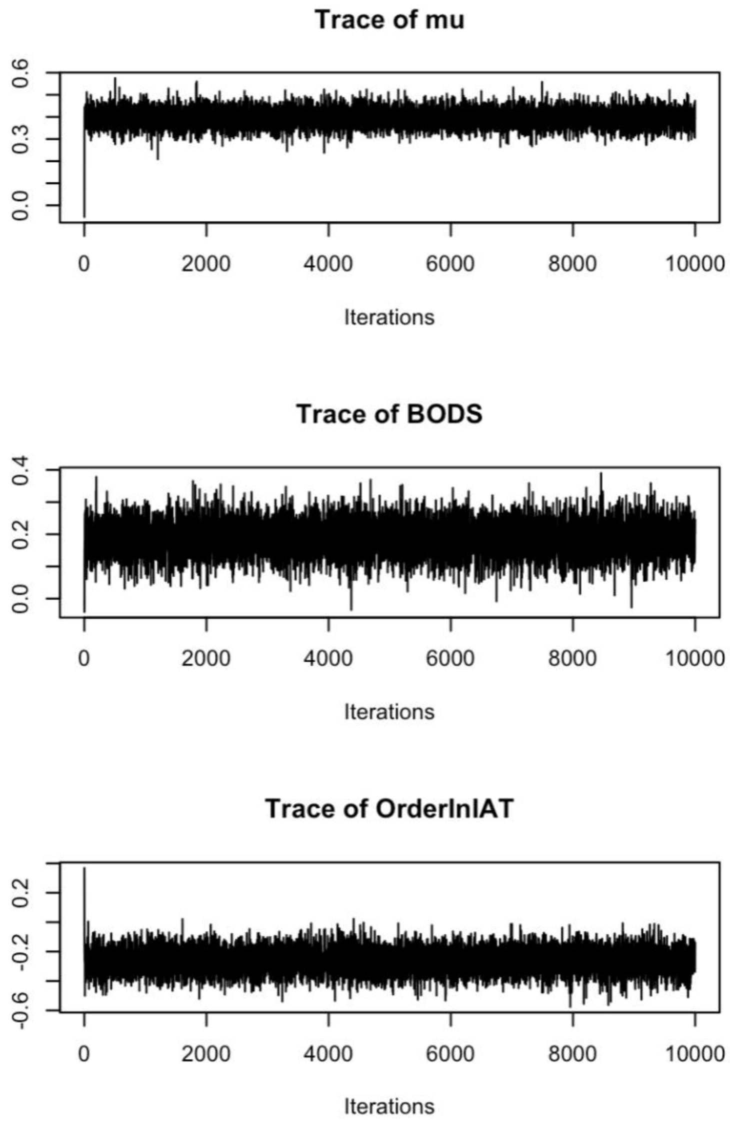

Figure S3. Chain diagnostics for the intercept (mu) and the predictors (BODS and the OrderInIAT) included in the winning model.

References

Bürkner, P. C. (2016). brms: An R package for Bayesian multilevel models using Stan. *Journal of Statistical Software*, 80(1), 1-28.

Liuzza, M. T., Lindholm, T., Hawley, C. B., Sendén, M. G., Ekström, I., Olsson, M. J., & Olofsson, J. K. (2018). Body odour disgust sensitivity predicts authoritarian attitudes. *Royal Society open science*, *5(2)*, 171091.
